# Comprehensive analysis of ZNF692 as a potential biomarker associated with immune infiltration in a pan cancer analysis and validation in hepatocellular carcinoma

**DOI:** 10.18632/aging.205218

**Published:** 2023-11-17

**Authors:** Hongjie Cai, Song Chen, Zhiqiang Wu, Fan Wang, Shuangyan Tang, Dongbing Li, Dongliang Wang, Wenbo Guo

**Affiliations:** 1 Department of Interventional Radiology, The First Affiliated Hospital of Sun Yat-Sen University, Guangzhou 510062, Guangdong, China; 2 Department of Minimally Invasive Interventional Therapy, Sun Yat-Sen University Cancer Center and Sun Yat-Sen University State Key Laboratory of Oncology in South China, Collaborative Innovation Center for Cancer Medicine, Guangzhou 510060, Guangdong, China; 3 Department of Translational Medicine, ChosenMed Technology (Beijing) Co., Ltd., Beijing 100176, China

**Keywords:** prognosis, diagnosis, genomic alterations, immune infiltration, drug sensitivity

## Abstract

Currently, the roles of ZNF692 have been documented exclusively in lung, colon, and cervical cancers. However, its involvement in pan cancer remains unknown. In this study, we employed bioinformatics analysis and experimental validation to investigate the role of ZNF692 in pan cancer. Our findings revealed aberrant expression of ZNF692 across various types of cancer. High expression of ZNF692 was associated with poor overall survival (OS) in ACC, COAD, KIRC, LAML, and LIHC. ZNF692 exhibited promising diagnostic potential in certain tumor types. A significant correlation was observed between high ZNF692 expression and advanced stages of ACC, BLCA, KICH, KIRC, LIHC, and OV. The expression of ZNF692 exhibited a significant association with microsatellite instability (MSI) in eight types of cancer and tumor mutational burden (TMB) in ten types of cancer. A noteworthy correlation was observed between ZNF692 expression and immune infiltration as well as immune checkpoints. Amplification of ZNF692 emerged as the most frequent alteration in pan cancer. ZNF692 was implicated in various biological processes, cellular components, and molecular functions within the context of pan cancer. It is plausible that ZNF692 may contribute to chemotherapy and potentially be linked to chemoresistance. We constructed a competing endogenous RNA (ceRNA) network involving AC009403.11/miR-126-3p/ZNF692 in hepatocellular carcinoma (HCC). The expression of ZNF692 exhibited a notable upregulation in HCC cell lines. Aberrant expression of ZNF692 was observed across various types of cancer. ZNF692 holds potential as a valuable diagnostic, prognostic, and therapeutic target in the context of pan cancer.

## INTRODUCTION

Cancer poses a significant public health obstacle in the pursuit of enhancing quality of life and prolonging lifespan [1]. In the year 2020, a staggering 19.3 million new cancer cases and approximately 10 million cancer-related deaths were reported globally [2]. Currently, the primary treatment modalities for cancer encompass surgical intervention, chemotherapy, radiotherapy, targeted therapy, and immunotherapy [3]. However, despite certain achievements in clinical practice, the emergence of drug resistance, adverse effects, and other complications contribute to suboptimal prognosis and survival rates among patients [4]. Therefore, we need to find new diagnostic and prognostic biomarkers to treat cancer.

Zinc finger protein 692 (ZNF692) was initially discovered as a transcription factor that interacts with the promoter element of phosphoenolpyruvate carboxykinase (PEPCK) and is located on chromosome 1q44 [5]. Its association with Wilms’ tumor recurrence has been established, and in clear cell renal cell carcinoma (ccRCC), ZNF692 facilitates proliferation by transcriptionally suppressing crucial genes [6, 7]. ZNF692 emerges as a novel oncogene and a promising therapeutic target for individuals diagnosed with colon adenocarcinoma (COAD) [8]. ZNF692 exhibited potential as an oncogene and biomarker in lung adenocarcinoma (LUAD) through its influence on cellular metabolism [9]. ZNF692 displayed promise as a therapeutic target and prognostic marker for cervical cancer (CC) [10]. Nevertheless, the precise role of ZNF692 in pan cancer remains uncertain.

This study investigates the expression, diagnostic significance, association with clinical characteristics and prognosis, genetic alterations, potential regulatory networks, correlation with immune infiltration and immune checkpoint genes, correlation with tumor mutational burden (TMB)/microsatellite instability (MSI), and drug sensitivity of ZNF692 across various cancer types. The results indicate that ZNF692 holds promise as a potential biomarker for tumor diagnosis, prognosis, and immunotherapeutic interventions.

## RESULTS

### Aberrant expression of ZNF692 in pan cancer

**Figure 1:**
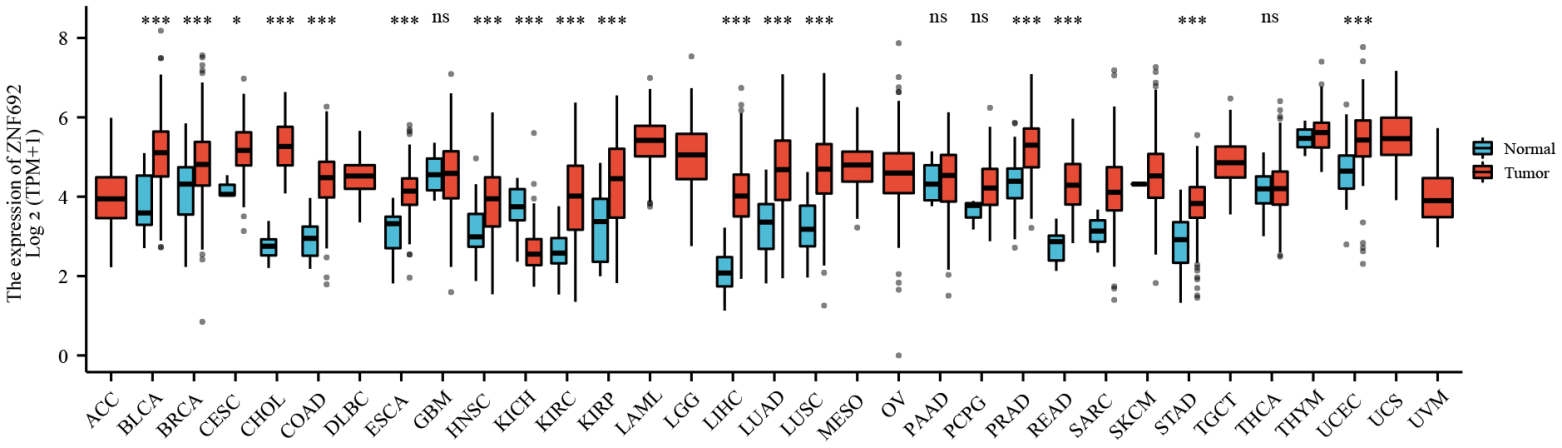
**Aberrant expression of ZNF692 in pan cancer.** Ns represents *p* > 0.05. * indicates *p* < 0.05. *** represents *p* < 0.001.

The results depicted in Figure 1 demonstrated a substantial upregulation of ZNF692 expression in multiple cancer types, including BLCA, BRCA, CESC, CHOL, COAD, ESCA, HNSC, KIRC, KIRP, LIHC, LUAD, LUSC, PRAD, READ, SKCM, STAD, and UCEC. Conversely, a significant downregulation of ZNF692 expression was observed in KICH. These findings suggest a potential correlation between ZNF692 and the pathogenesis and advancement of these malignancies.

### Expression of ZNF692 in pan cancer correlated with prognosis

**Figure 2:**
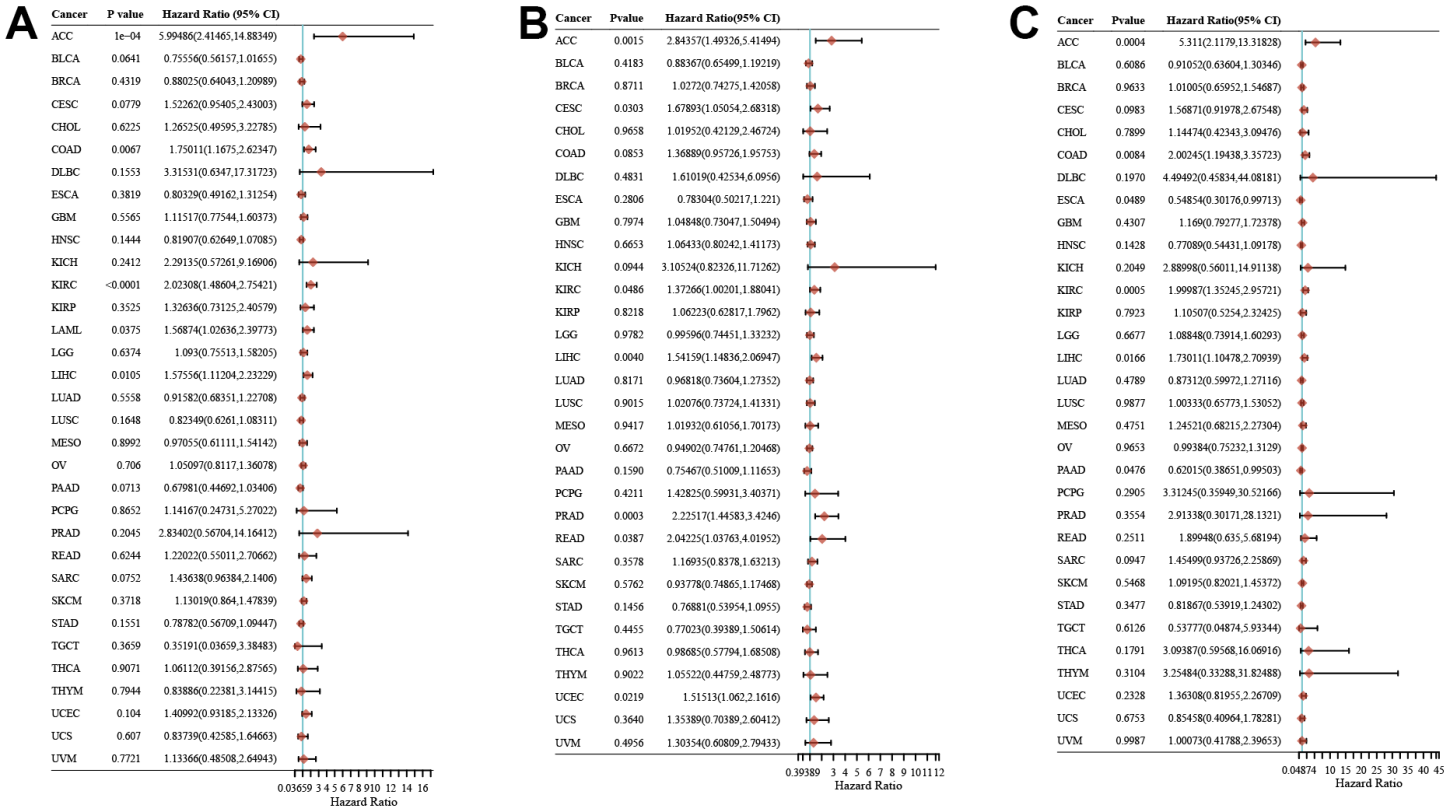
**The expression of ZNF692 in pan cancer was associated with prognosis.** (**A**) Overall survival. (**B**) Progression free survival. (**C**) Disease specific survival.

The findings illustrated in Figure 2A demonstrated a statistically significant association between the expression of ZNF692 and unfavorable overall survival (OS) in several cancer types, including ACC (*p* < 0.001), COAD (*p* = 0.0067), KIRC (*p* < 0.0001), LAML (*p* = 0.0375), and LIHC (*p* = 0.0105). The results presented in Figure 2B indicated a significant association between ZNF692 and unfavorable progression-free survival (PFS) in several cancer types, including ACC (*p* = 0.0015), CESC (*p* = 0.0303), KIRC (*p* = 0.0486), LIHC (*p* = 0.0040), PRAD (*p* = 0.0003), READ (*p* = 0.0387), and UCEC (*p* = 0.0219). The results presented in Figure 2C indicate a statistically significant correlation between ZNF692 expression and disease-specific survival (DSS) outcomes in several cancer types, including ACC (*p* = 0.0004), COAD (*p* = 0.0084), KIRC (*p* = 0.0005), and LIHC (*p* = 0.0166). Conversely, ZNF692 expression were significantly associated with favorable DSS in ESCA (*p* = 0.0489) and PAAD (*p* = 0.0476).

### The diagnostic value of ZNF692 in pan cancer

**Figure 3:**
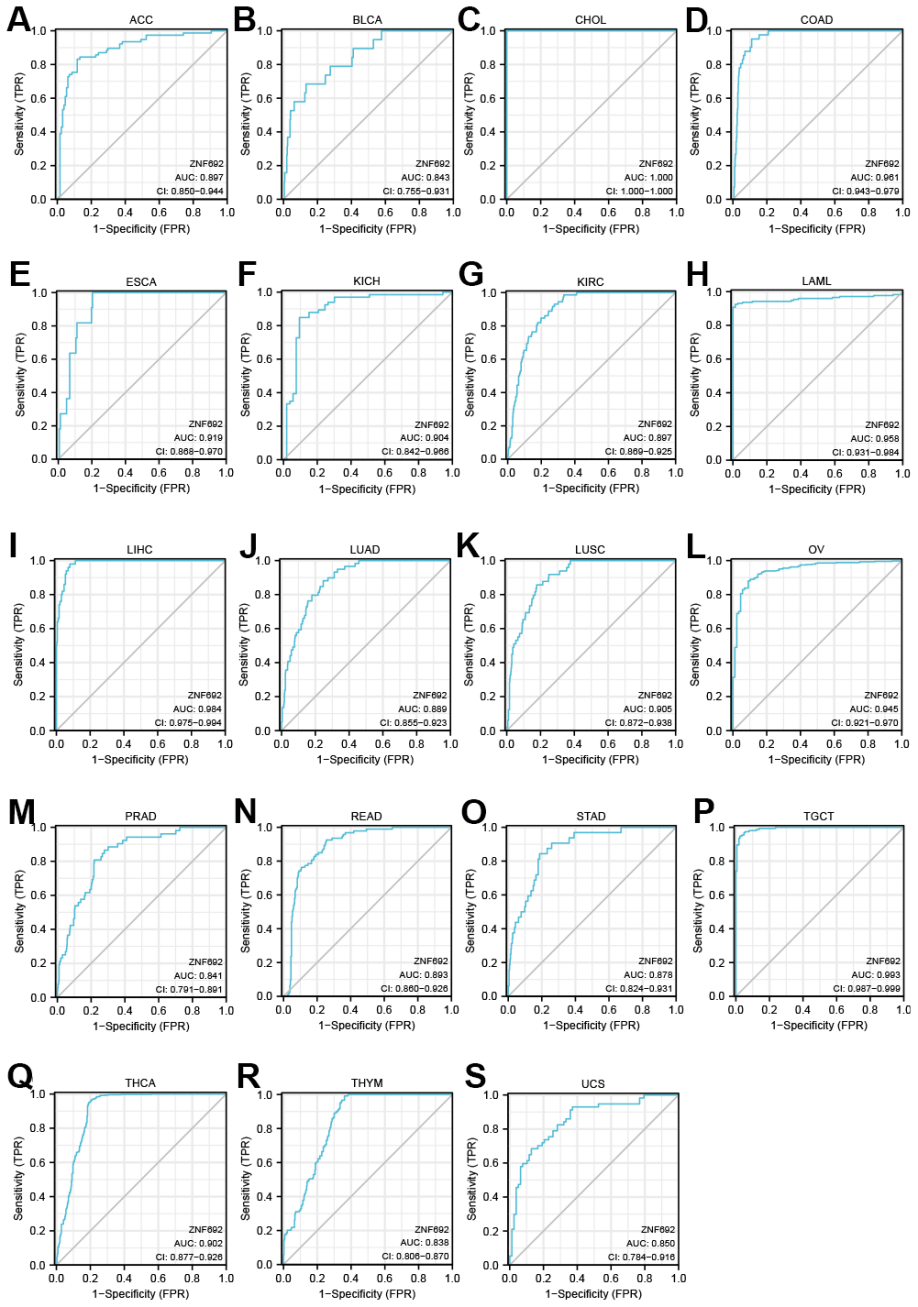
**ROC curves showed that ZNF692 had a high diagnostic value (AUC>0.8) in several types of cancers.** (**A**) ACC. (**B**) BLCA. (**C**) CHOL. (**D**) COAD. (**E**) ESCA. (**F**) KICH. (**G**) KIRC. (**H**) LAML. (**I**) LIHC. (**J**) LUAD. (**K**) LUSC. (**L**) OC. (**M**) PRAD. (**N**) READ. (**O**) STAD. (**P**) TGCT. (**Q**) THCA. (**R**) THYM. (**S**) UCS.

ROC curves were utilized to assess the diagnostic efficacy of ZNF692 in 33 tumors. The analysis of the ROC curves revealed the presence of the ZNF692 gene in various tumor types, including ACC (AUC = 0.897), BLCA (AUC = 0.843), CHOL (AUC = 1.000), COAD (AUC = 0.961), ESCA (AUC = 0.919), KICH (AUC = 0.904), KIRC (AUC = 0.897), LAML (AUC = 0.958), LIHC (AUC = 0.984), LUAD (AUC = 0.889), LUSC (AUC = 0.905), OV (AUC = 0.945), PRAD (AUC = 0.841), READ (AUC = 0.893), STAD (AUC = 0.878), TGCT (AUC = 0.993), THCA (AUC = 0.902), THYM (AUC = 0.838), and UCS (AUC = 0.850) (Figure 3).

### ZNF692 expression in pan cancer correlates with stage, TMB, and MSI

**Figure 4:**
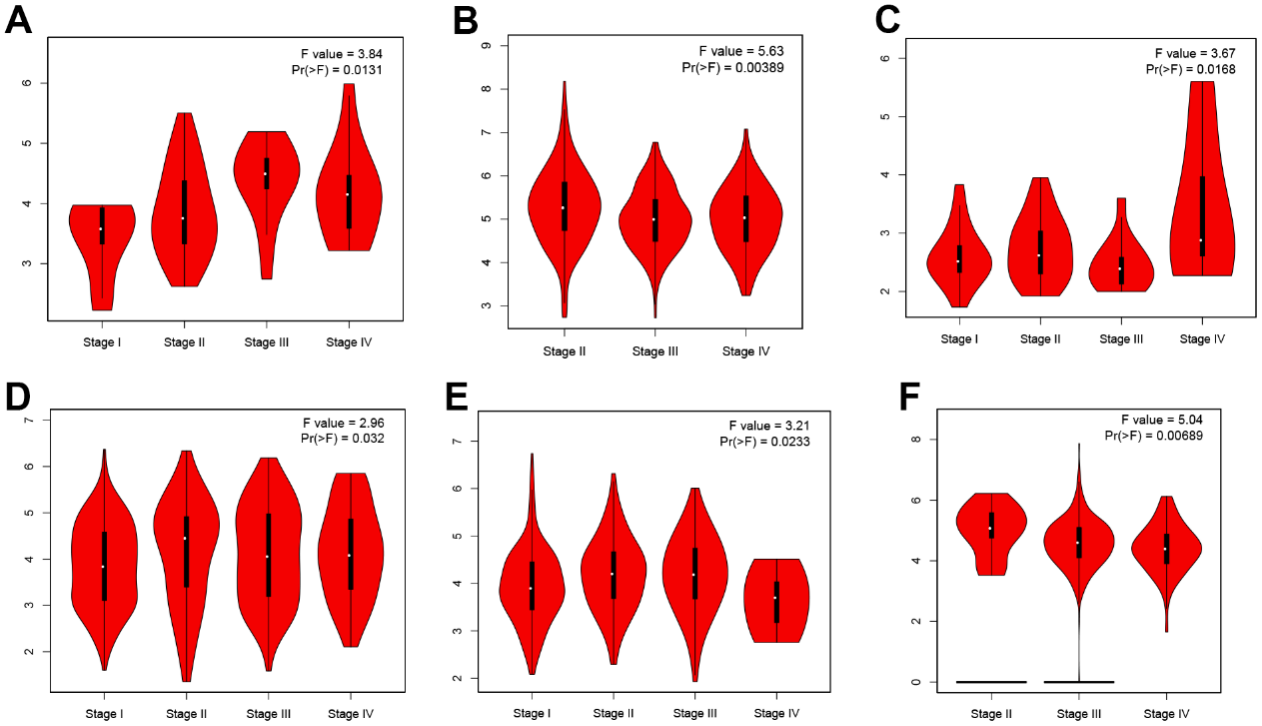
**The expression of ZNF692 in pan cancer is associated with stage.** (**A**) ACC. (**B**) BLCA. (**C**) KICH. (**D**) KIRC. (**E**) LICH. (**F**) OC.

**Figure 5:**
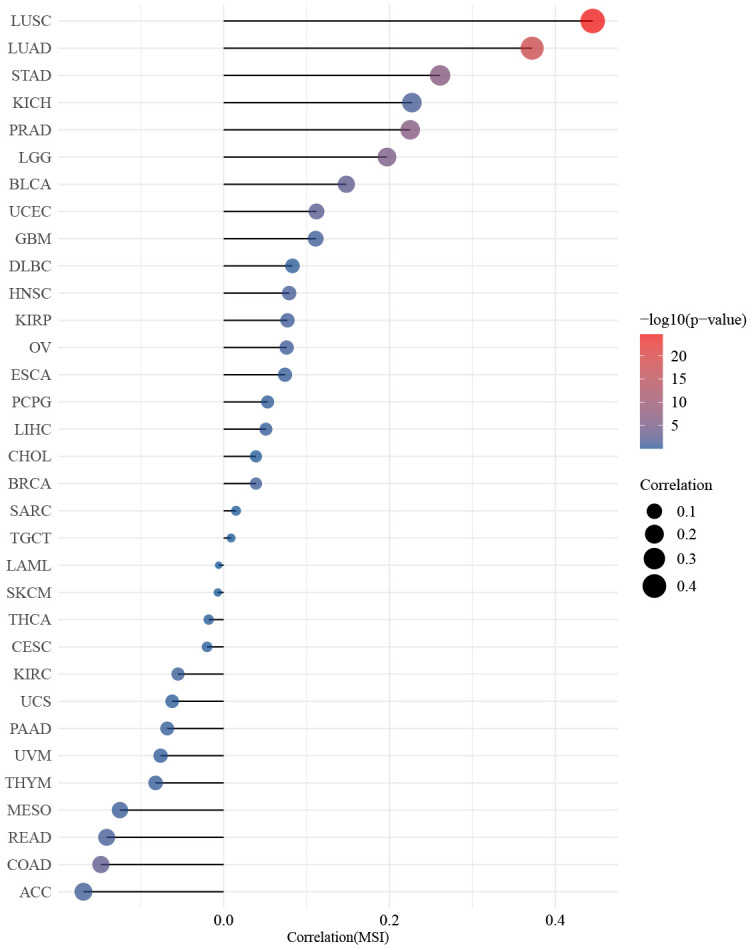
Spearman analysis showed that the expression of ZNF692 in pan cancer was associated with MSI.

**Figure 6:**
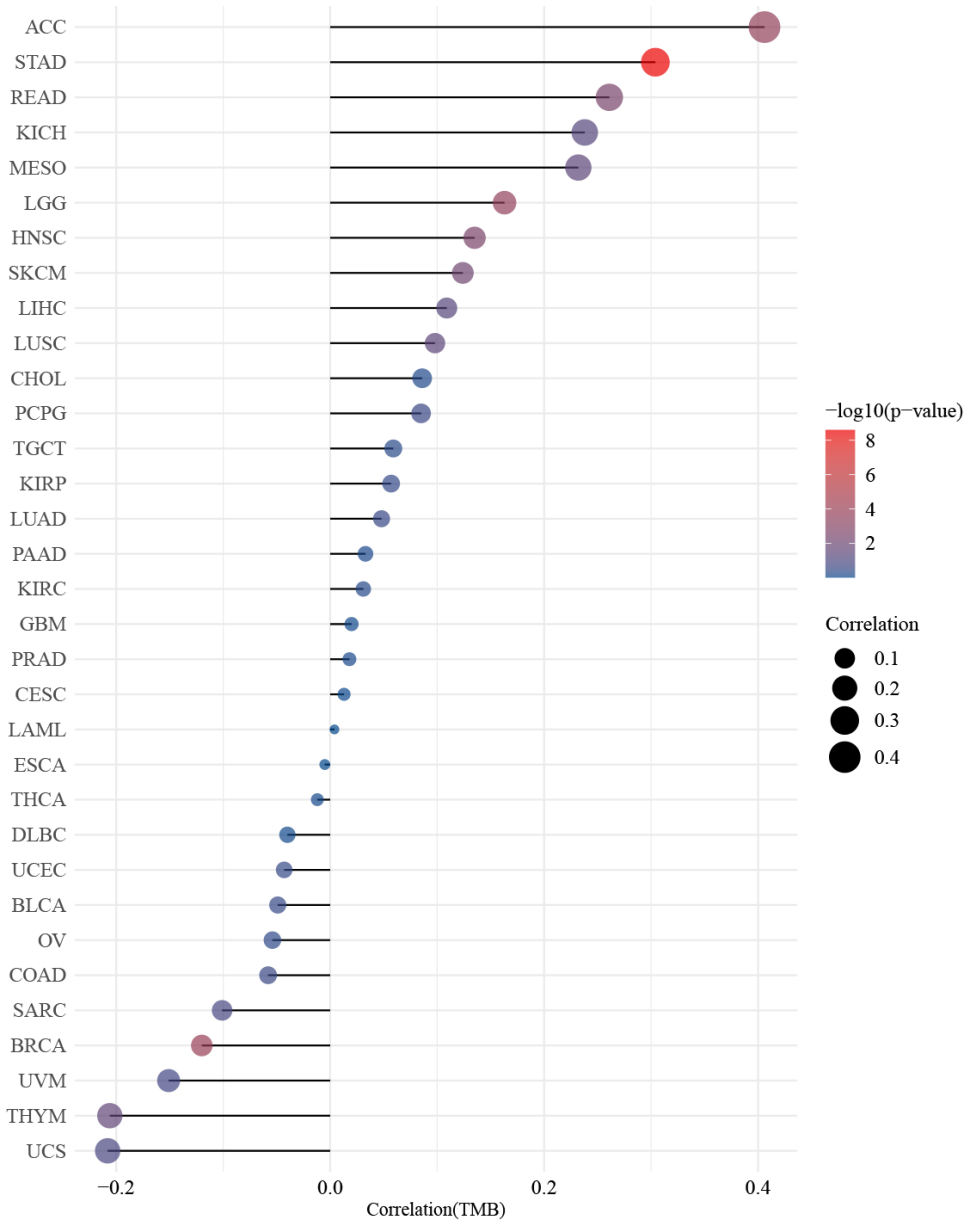
Spearman analysis showed that the expression of ZNF692 in pan cancer was associated with TMB.

The findings presented in Figure 4 demonstrated a statistically significant relationship between ZNF692 and the stages of several types of cancer, including ACC, BLCA, KICH, KIRC, LIHC, and OV. The results depicted in Figure 5 revealed a significant positive correlation between the expression of ZNF692 and the MSI status of BLCA (*p* = 0.003), LGG (*p* < 0.001), LUAD (*p* < 0.001), LUSC (*p* < 0.001), PRAD (*p* < 0.001), STAD (*p* < 0.001), and UCEC (*p* < 0.001), while a negative correlation was observed with the MSI status of COAD (*p* = 0.002). According to the findings presented in Figure 6, there was a significant positive correlation observed between ZNF692 expression and the TMB status of ACC (*p* < 0.001), HNSC (*p* = 0.003), LGG (*p* < 0.001), LUSC (*p* = 0.035), MESO (*p* = 0.037), READ (*p* = 0.004), SKCM (*p* = 0.008), and STAD (*p* < 0.001), Conversely, a significant negative correlation was found between ZNF692 expression and the TMB status of ACC (*p* < 0.001) and THYM (*p* = 0.026).

### The genetic alterations of ZNF692 in pan cancer

**Figure 7:**
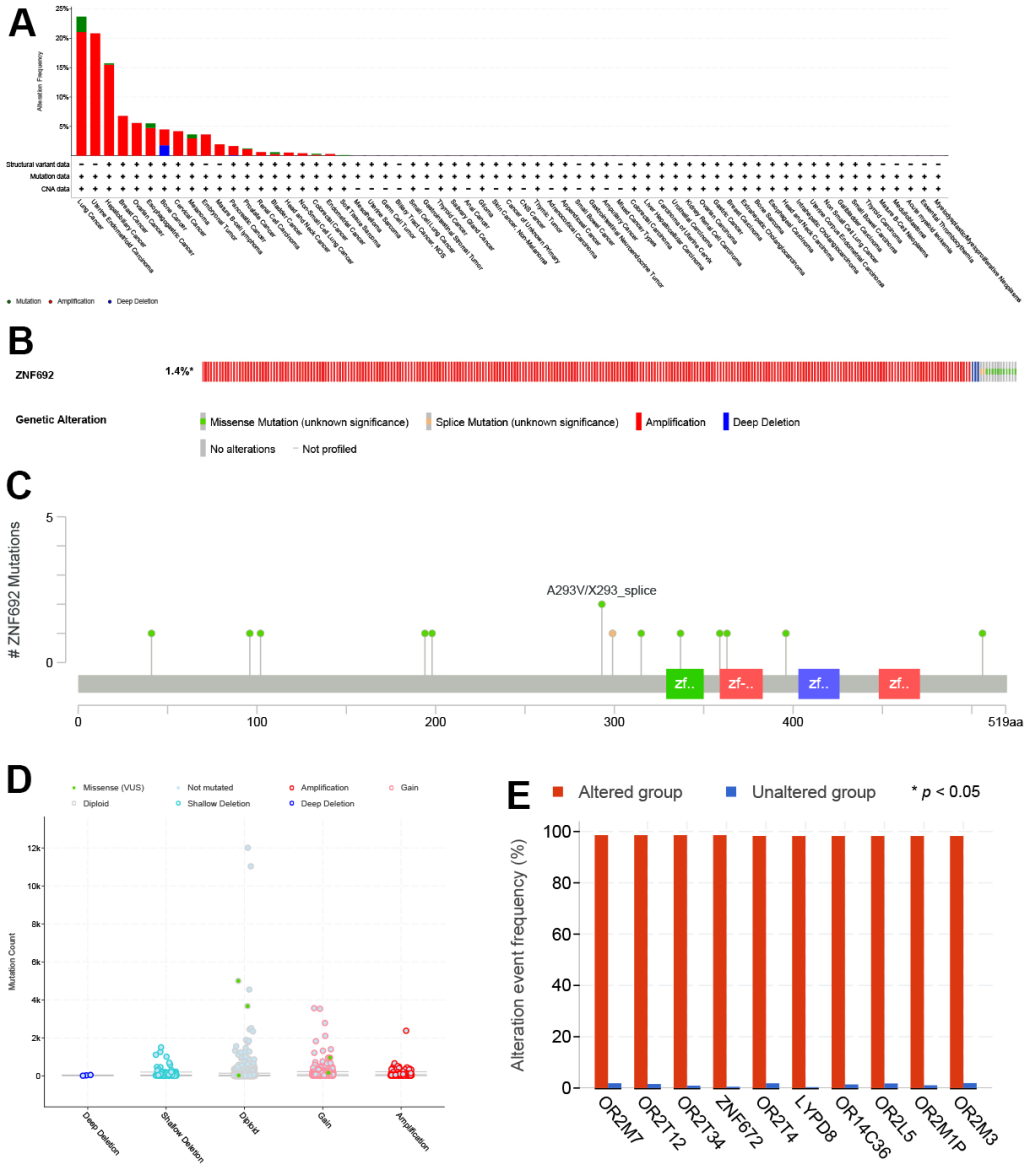
**Genetic alterations of ZNF692 in pan cancer.** (**A**) Summary of ZNF692 gene alterations. (**B**) Frequency of ZNF692 gene alterations. (**C**) Description of ZNF692 gene alterations. (**D**) Type of ZNF692 gene alterations. (**E**) Top 10 genes commonly altered in the ZNF692 alteration group.

Genetic alterations in ZNF692 were identified across multiple cancer types, including lung cancer (amplification 21.05%; mutation 2.63%), uterine endometrioid carcinoma (amplification 20.83%), hepatobiliary cancer (amplification 15.51%; mutation 0.22%), breast cancer (amplification 6.78%); ovarian cancer (amplification 5.57%), esophagogastric cancer (amplification 4.72%; mutation 0.79%), bone cancer (amplification 2.68%; deep deletion 1.79%), cervical cancer (amplification 4.17%), mature B-cell lymphoma (amplification 1.94%), pancreatic cancer (amplification 1.57%; deep deletion 0.08%), prostate cancer (amplification 1.12%; mutation 0.11%), renal cell carcinoma (amplification 0.62%), bladder cancer (mutation 0.3%; amplification 0.3%), head and neck cancer (amplification 0.53%), non-small cell lung cancer (amplification 0.41%), colorectal cancer (mutation 0.17%; amplification 0.17%), endometrial cancer (amplification 0.29%), and soft tissue sarcoma (mutation 0.11%) (Figure 7A). The predominant genetic alterations observed in ZNF692 were amplification, missense mutation, deep deletion, and splice mutation, as depicted in Figure 7B. As shown in Figure 7C, mutations in ZNF692 in pan cancer included 12 missense mutations (S363C, E198K, E194K, L41F, L96P, A293V, P506L, F359L, R396L, R337G, G102E, and G315V) and 2 splice site mutations (X293_splice and X299_splice). Figure 7D illustrated that the prevalent putative copy number alterations for ZNF692 included deep deletion, shallow deletion, diploid, gain, and amplification. The altered group exhibited a higher likelihood of being affected due to the gene alterations in OR2M7, OR2T12, OR2T34, ZNF672, OR2T4, LYPD8, OR14C36, OR2L5, OR2M1P, and OR2M3 (Figure 7E).

### ZNF692 expression in pan cancer correlated with immune infiltration and immune checkpoints

**Figure 8:**
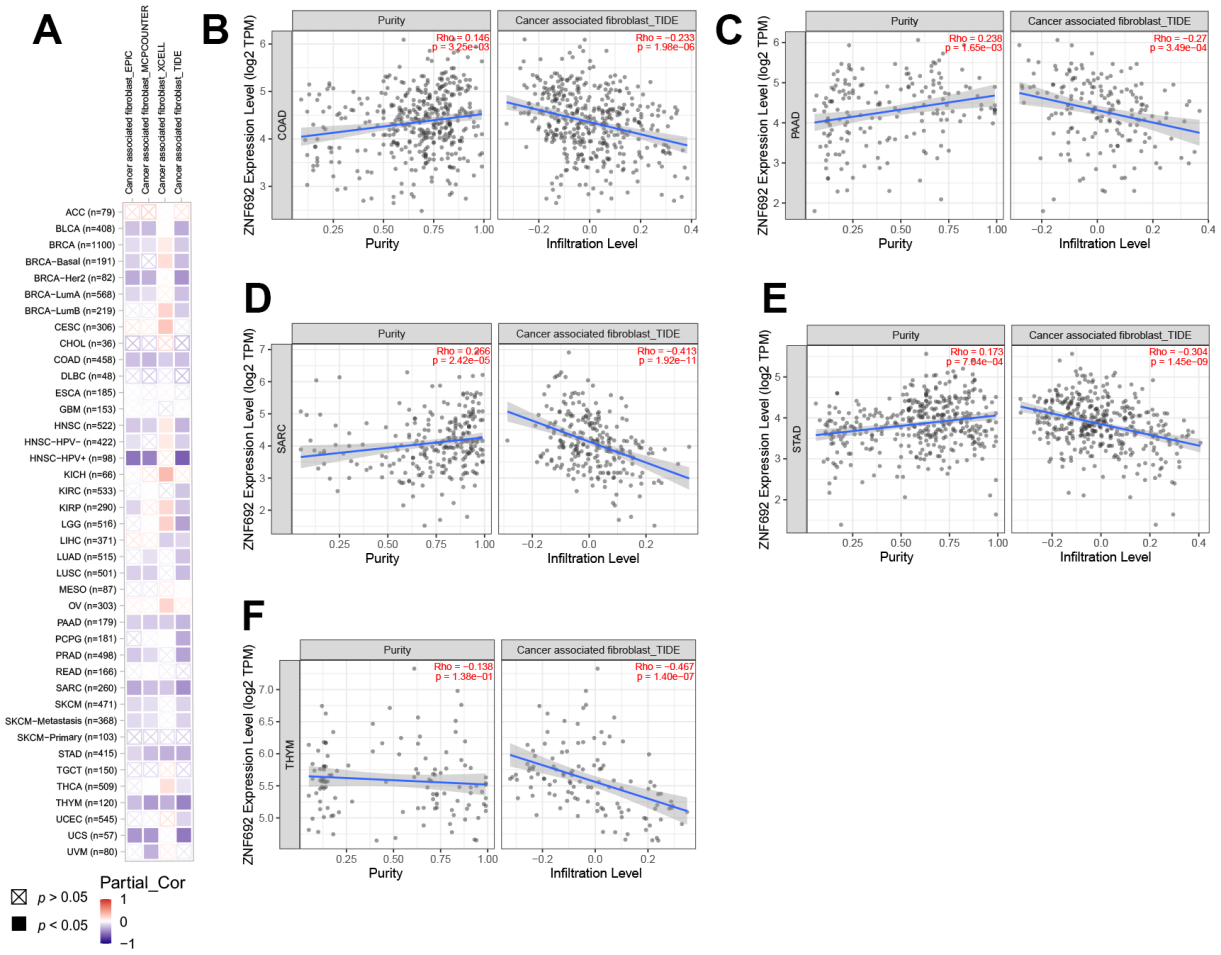
**The expression of ZNF692 was associated with cancer-associated fibroblasts in pan cancer.** (**A**) Correlation of ZNF692 expression with CAF (TIMER2.0). (**B**) COAD. (**C**) PAAD. (**D**) SARC. (**E**) STAD. (**F**) THYM.

**Figure 9:**
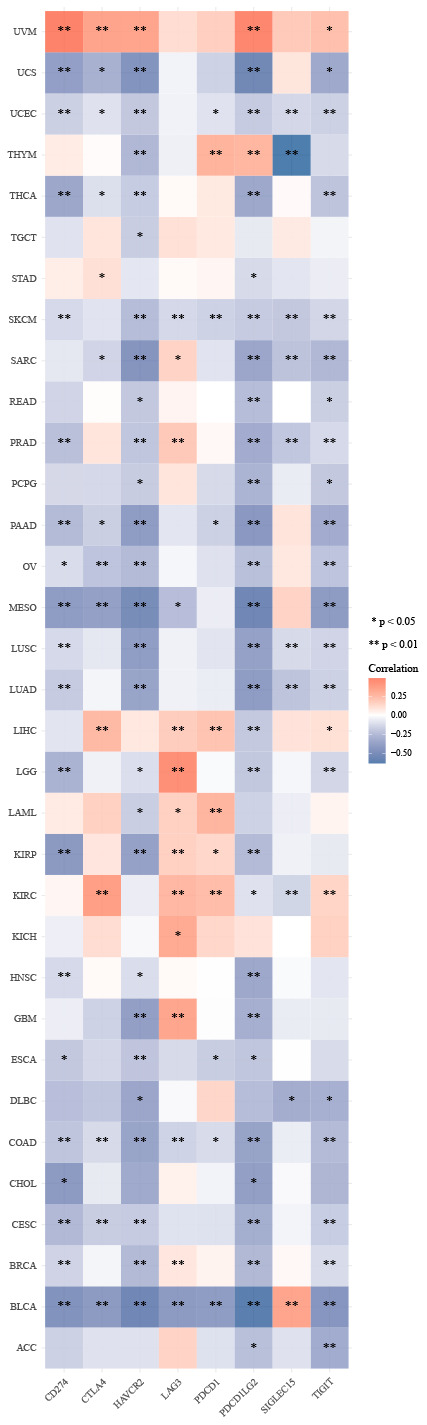
**The expression of ZNF692 is associated with immune checkpoint genes in pan cancer.** * indicates *p* < 0.05. ** represents *p* < 0.01.

The present study provides evidence of a positive correlation between the expression of ZNF692 and cancer-associated fibroblasts (CAF) in specific tumor types, as depicted in Figure 8A. The results obtained from the TIDE algorithm, as illustrated in Figure 8B–8F, demonstrate a significant and positive association between ZNF692 expression and CAF in COAD, PAAD, SARC, STAD, and THYM. The findings reveal a significant correlation between the expression levels of ZNF692 and immune checkpoint genes in 33 different tumor types, as depicted in Figure 9. The expression levels of ZNF692 in various cancer types, including BLCA, BRCA, GBM, KIRC, KIRP, LAML, LGG, LIHC, PRAD, SARC, STAD, and THYM, were found to be correlated with the expression levels of immune checkpoint genes. The expression of ZNF692 in KICH and UVM showed a positive correlation with the expression of immune checkpoint genes. Conversely, the expression of ZNF692 in ACC, CESC, CHOL, COAD, DLBC, ESCA, HNSC, LUAD, LUSC, MESO, OV, PRAD, PCPG, READ, SKCM, TGCT, THCA, UCEC, and UCS exhibited a negative correlation with the expression of immune checkpoint genes. These findings suggested that ZNF692 may play a role in mediating the immune response to pan cancer by regulating these immune checkpoint genes.

### ZNF692 related pathways in pan cancer

**Figure 10:**
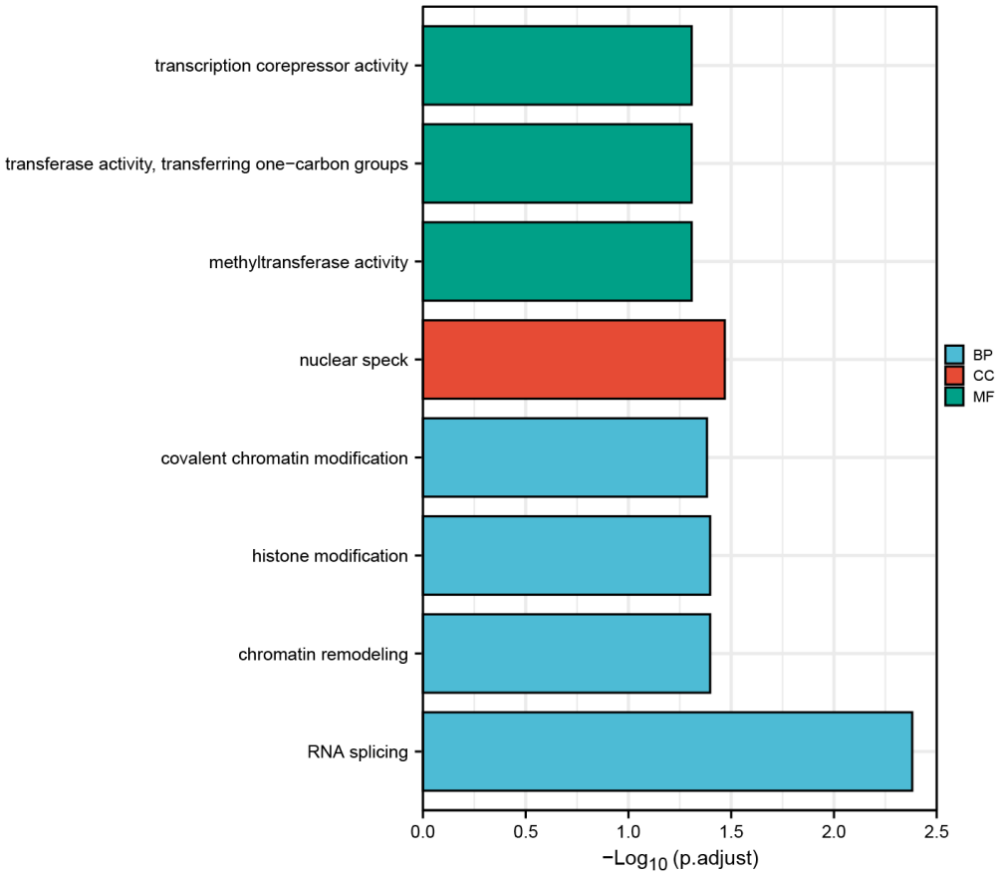
Pathway enrichment analysis of ZNF692 and its related genes.

According to the findings presented in Figure 10, the study revealed the presence of four significant biological processes, namely, RNA splicing, chromatin remodeling, histone modification, and covalent chromatin modification. Additionally, one relevant cellular component, specifically nuclear speck, and three pertinent molecular functions, including methyltransferase activity, transferase activity involving one-carbon groups, and transcription corepressor activity, were identified.

### ZNF692 is associated with drug sensitivity

The expression of ZNF692 exhibited a positive correlation with the drug sensitivity of chlorobisoximatobismuth(iii)-dihydrochloride, chlorotris(chinoline-8-olato) titanium(iv)-trihydrochloride, tetrakis(8-chinolineolato) titanium(iv)-tetrahydrochloride, benzoxazole, 2-[(1-methyl-4-nitro-1h-imidazol-5-yl)thio]-, and antineoplastic-637578, as indicated in Supplementary Table 1. The expression of ZNF692 exhibited a negative correlation with the drug sensitivity of various compounds, including 1-naphthalenecarboxamide, n,n’-1,8-(octanediyl)bis-, N-cyclohexyl-4-((1-phenyl-1H-pyrazolo[3,4-d]pyrimidin-4…, 4-piperidinone, 3,5-dimethyl-1-nitroso-2,6-diphenyl-, (E)-5-chloro-3-((6-(4-chlorophenyl)-2-cyclopropylimidaz…, and 1,3-diphenyl-4-(3-phenyl-4,5-dihydro-1H-pyrazol-5-yl)-1… (refer to Supplementary Table 1). ZNF692 may mediate resistance to certain drugs in pan cancer.

### ZNF692 expression in single cells correlated with tumor characteristics

**Figure 11:**
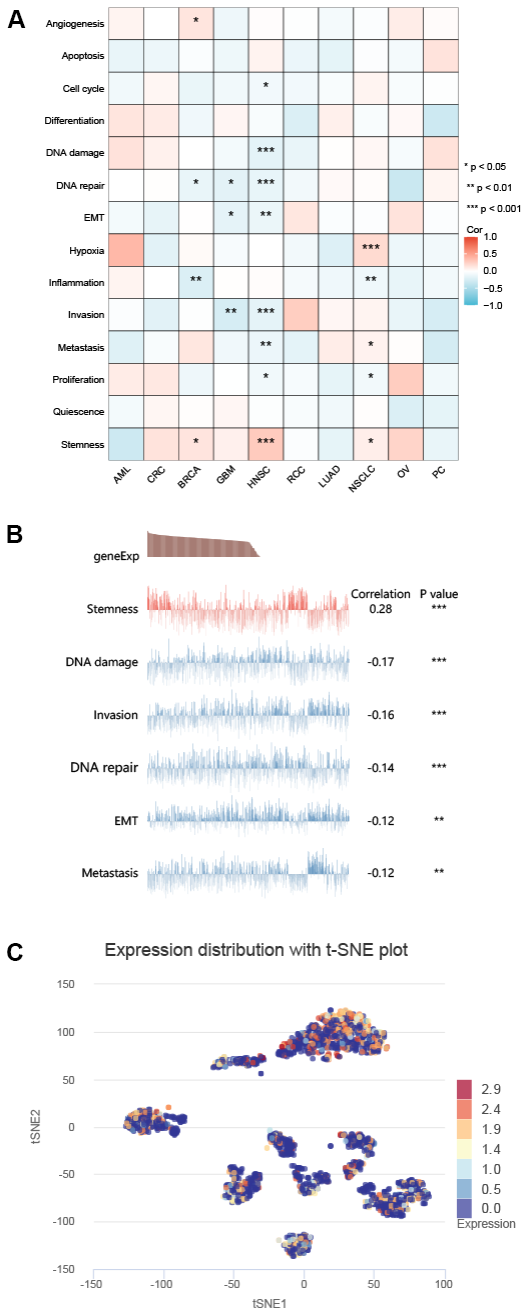
**CancerSEA showed that the expression of ZNF692 in single cell sequencing was associated with tumor functional status.** (**A**) The expression of ZNF692 was associated with different tumors. (**B**) The expression of ZNF692 in GBM was associated with different functional states. (**C**) ZNF692 expression in GBM single cells (T-SNE). * indicates *p* < 0.05. ** represents *p* < 0.01. *** *p* < 0.001.

The results depicted in Figure 11A demonstrated that ZNF692 expression in BRCA exhibited a positive correlation with angiogenesis and stemness, but a negative correlation with DNA repair and inflammation. Conversely, ZNF692 expression in GBM displayed a negative correlation with DNA repair, EMT, and invasion. In HNSC, ZNF692 expression exhibited a positive correlation with stemness, while displaying a negative correlation with cell cycle, DNA damage, DNA repair, EMT, invasion, metastasis, and proliferation. Additionally, ZNF692 expression in NSCLC demonstrated a positive correlation with hypoxia, metastasis, and stemness, but a negative correlation with inflammation and proliferation. The results depicted in Figure 11B indicated a significant correlation between ZNF692 expression in HNSC and various cellular processes, including stemness, DNA damage, invasion, DNA repair, EMT, and metastasis. Additionally, Figure 11C showcased the expression of ZNF692 in individual cells of HNSC through T-SNE plots. These findings suggested a potential involvement of ZNF692 in the genesis and progression of cancer.

### CeRNA network of ZNF692 in HCC

**Figure 12:**
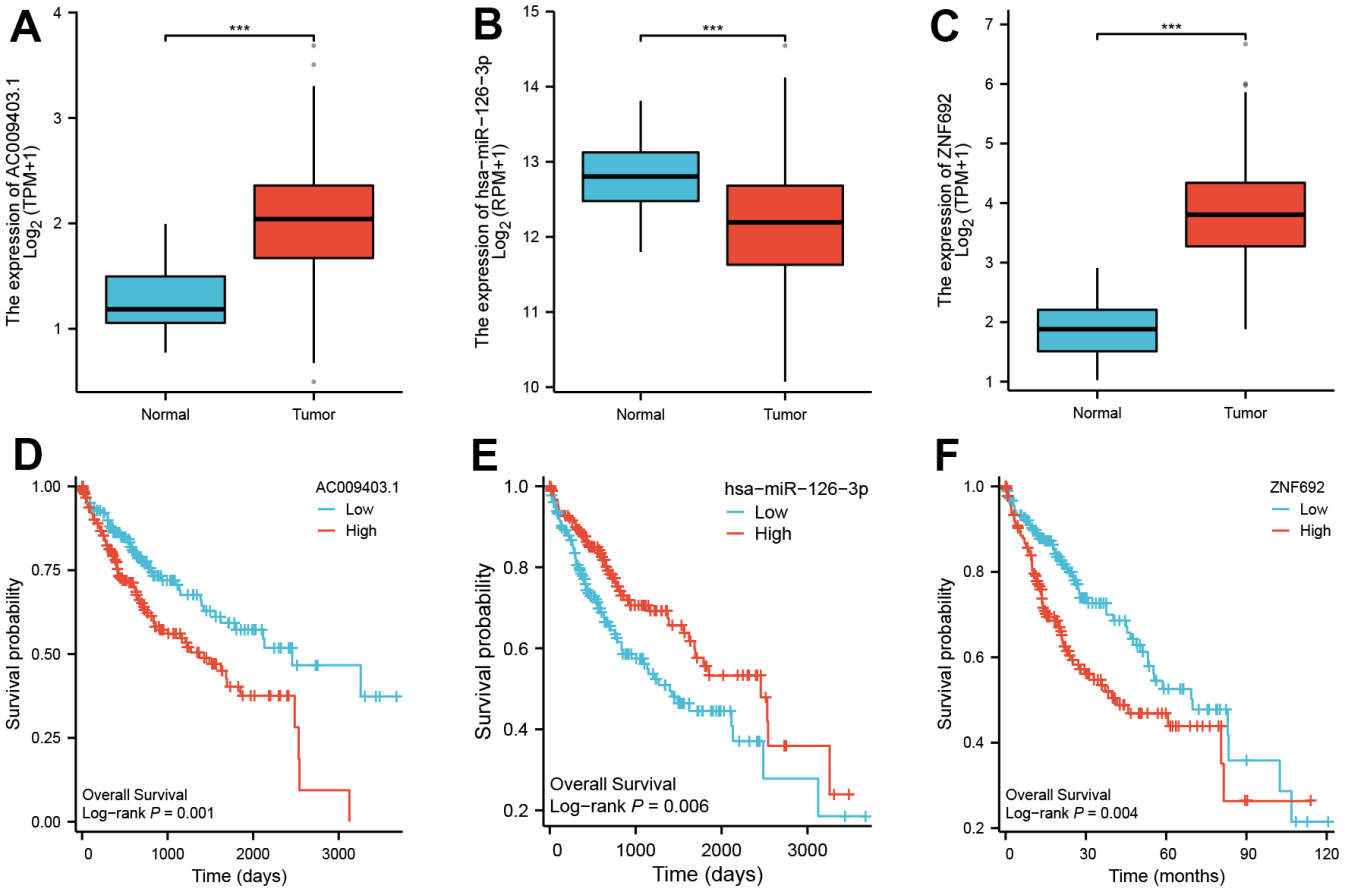
Possible ceRNA network of ZNF692 in HCC.

**Figure 13:**
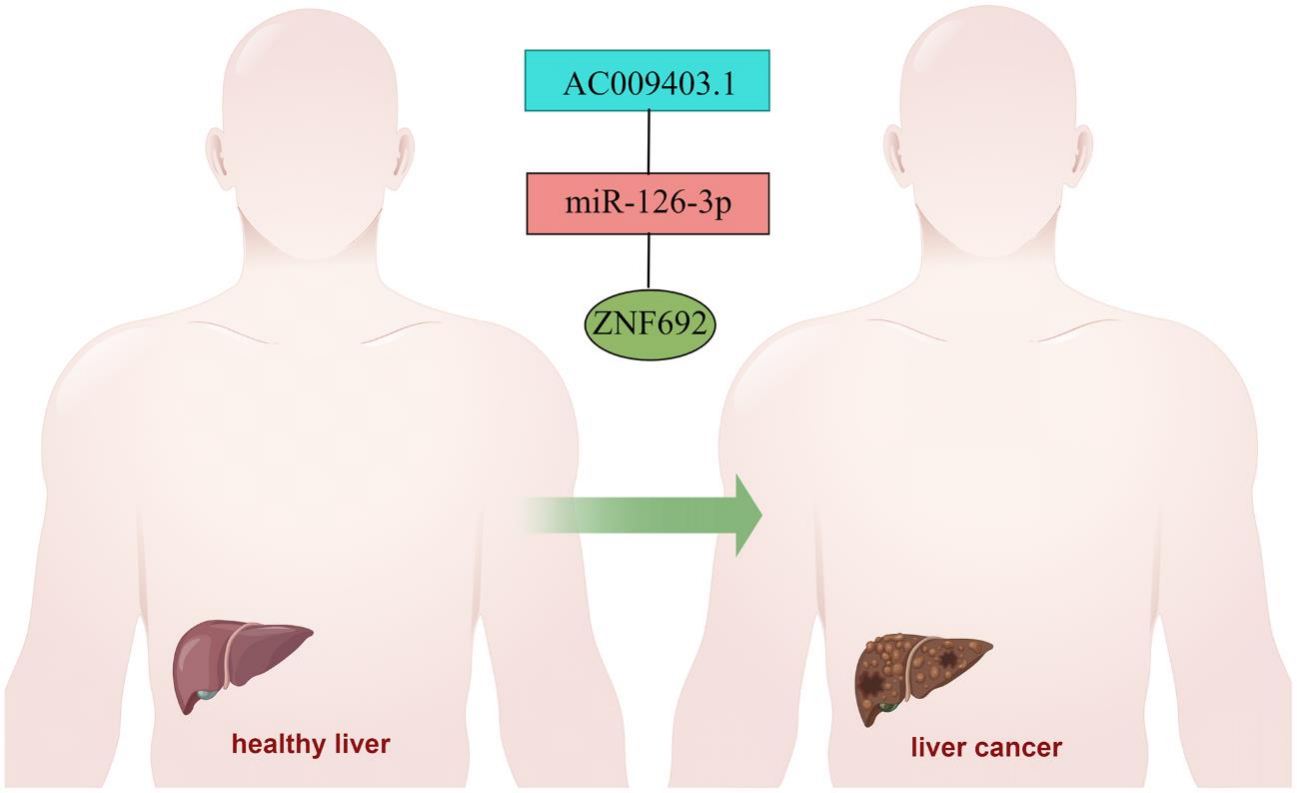
ZNF692 mediated the ceRNA network of HCC genesis.

The expression of ZNF692 exhibited a significant upregulation in HCC. However, the regulatory network associated with ZNF692 remained unidentified. Through analysis of the TCGA database, we discovered a significant upregulation of AC009403.1 expression in HCC (as depicted in Figure 12A), which was also indicative of a poor prognosis (*p* = 0.001, Figure 12D). The expression of miR-126-3p was found to be significantly downregulated in HCC (Figure 12B), and its low expression was associated with a poor prognosis (*p* = 0.006, Figure 12E). The upregulation of ZNF692 in HCC was found to be statistically significant (Figure 12C), and its high expression was indicative of a negative prognosis (*p* = 0.004, Figure 12F). To illustrate this, we employed Figdraw to construct a ceRNA network involving AC009403.1/miR-126-3p/ZNF692 in HCC (Figure 13).

### ZNF692 was significantly upregulated in HCC cell lines

**Figure 14:**
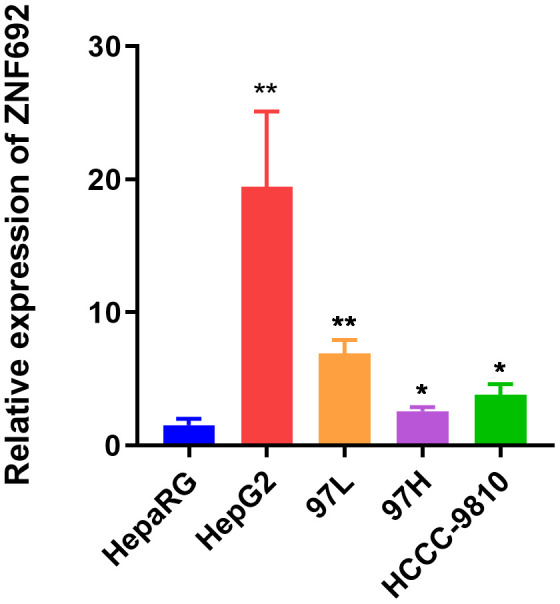
Expression of ZNF692 in normal hepatic epithelial cell HepaRG, and HCC lines HepG2, 97L, 97H, and HCCC-9810.

According to the data presented in Figure 14, the expression of ZNF692 exhibited a notable upregulation in various HCC cell lines, namely, HepG2 (*p* = 0.0055), 97L (*p* = 0.0011), 97H (*p* = 0.0299), and HCCC-9810 (*p* = 0.0141), when compared to the normal hepatic epithelial cell line HepaRG.

## DISCUSSION

Pan cancer analysis is employed to enhance the comprehension of the fundamentally disrupted molecules in pan cancer, thereby facilitating the identification of biomarkers for timely cancer detection and targeted therapeutic interventions [11]. TCGA contained multi-omics data for 33 tumors, allowing exploration of genetic alterations in different cancer types [11].

ZNF692 exhibited significant upregulation in ccRCC, LUAD, COAD, and CC [7–10]. However, the expression of ZNF692 in the context of pan cancer remains unknown. Consequently, this study aimed to investigate the potential role of ZNF692 in pan cancer. Our findings revealed aberrant expression of ZNF692 across various types of cancer, which aligns with previously reported data. Furthermore, we observed a significant upregulation of ZNF692 in HCC cell lines. Subsequently, we examined the prognostic and diagnostic value of ZNF692 expression in pan cancer. Notably, high expression levels of ZNF692 in ACC, COAD, KIRC, LAML, and LIHC were indicative of poor OS. The high expression of ZNF692 in several cancer types, including ACC, CESC, KIRC, LIHC, PRAD, READ, and UCEC, was indicative of poor PFS. Additionally, the high expression of ZNF692 in ACC, COAD, KIRC, and LIHC was associated with poor DSS. Notably, among the 19 analyzed cancer types, such as ACC, BLCA, CHOL, COAD, ESCA, KICH, KIRC, LAML, LIHC, LUAD, LUSC, OV, PRAD, READ, STAD, TGCT, THCA, THYM, and UCS, ZNF692 demonstrated high accuracy (AUC > 0.8) in predicting both tumor and normal outcomes.

No genetic alterations of ZNF692 have been reported in pan cancer. Through the utilization of the cBioPortal database, we have determined that amplification is the prevailing alteration of ZNF692 in pan cancer. Furthermore, we have identified co-alterations of OR2M7, OR2T12, OR2T34, ZNF672, OR2T4, LYPD8, OR14C36, OR2L5, OR2M1P, and OR2M3 within the ZNF692 alteration group.

In this study, we investigated the association between the expression of ZNF692 and various factors including stage, MSI, and TMB in pan cancer. Our findings revealed that the abnormal expression of ZNF692 was significantly correlated with stage in six tumors. Additionally, we observed a correlation between ZNF692 expression and MSI in eight cancer types, as well as with TMB in ten cancer types. However, further comprehensive investigation is required to fully understand the clinical significance of ZNF692.

Currently, there is limited knowledge regarding the involvement of ZNF692 in the human immune system. The tumor microenvironment (TME) is primarily composed of CAF [12]. As the effectiveness of immune checkpoint inhibitors (ICIs) relies on the tumor immune microenvironment, it is imperative to understand the immune landscape of HCC in order to appropriately select ICIs [13]. Consequently, we investigated the association between ZNF692 expression and immune infiltration as well as immune checkpoint expression across various types of cancer. Our findings indicate a correlation between ZNF692 expression in pan cancer and the infiltration of CAF, as well as the expression of eight immune checkpoint genes. The specific mechanism of ZNF692-mediated immune response needs to be further investigated.

ZNF692 was found to activate the PI3K/AKT pathway, thereby promoting growth and metastasis in COAD [8]. Upregulation of ZNF692 enhances G1/S conversion by regulating the p27 kip1/P Thr160-CDK2 signaling pathway in CC cells [10]. In this study, we found that ZNF692 is involved in the biology processes (RNA splicing, chromatin remodeling, histone modification, and covalent chromatin modification), cellular components (nuclear speck) and molecular functions (methyltransferase activity, transferase activity, transferring one-carbon groups, and transcription corepressor activity). The detailed mechanism of ZNF692-mediated molecular function needs to be further investigated.

However, the relationship between ZNF692 expression and drug sensitivity remains unclear. To investigate this, we utilized the RNAactDrug database and discovered that ZNF692 is associated with the sensitivity of various drugs. The mechanism of ZNF692-mediated resistance to some drugs needs further study.

However, our study encountered certain limitations. Firstly, we conducted a preliminary investigation into the clinical significance of ZNF692 in pan cancer, necessitating further validation through a larger sample size in future studies. Secondly, we conducted a preliminary exploration of the potential regulatory network of ZNF692 in pan cancer, which required verification through additional experiments in the future. Lastly, we constructed a ceRNA network involving AC009403.1/miR-126-3p/ZNF692 in HCC, but further experiments were required to validate our findings.

In summary, the potential of ZNF692 as a diagnostic and prognostic indicator in pan cancer is noteworthy. ZNF692 plays a crucial role in the initiation and advancement of tumors by modulating MSI, TMB, CAF infiltration, immune checkpoint inhibitors, and drug responsiveness. This investigation sheds light on the diverse functions of ZNF692 in pan cancer and presents a compelling justification for considering ZNF692 as an innovative therapeutic strategy.

## MATERIALS AND METHODS

### Expression analysis of ZNF692 in pan cancer

RNAseq data were obtained and compiled from the TCGA database (https://portal.gdc.cancer.gov) using the STAR process for a total of 33 tumor projects. The data were extracted in TPM format and subsequently subjected to log2 transformation (value+1). Statistical analysis was conducted using the appropriate statistical methods, namely, the stats package and car package, taking into consideration the characteristics of the data format. In instances where the statistical requirements were not met, statistical analysis was not performed. The resulting data were visualized using the ggplot2 package [14]. The subgroups encompassed a diverse range of 33 tumor types [11, 15].

### Analysis of the correlation between ZNF692 and prognosis in pan cancer

Univariate Cox regression analysis was conducted using the ‘forestplot’ R package to display the P-value, hazard ratio (HR), and 95% confidence interval (CI) for each variable. RNA sequencing expression (level 3) profiles and corresponding clinical information for 33 tumors were obtained from the TCGA [16–18]. The ‘forestplot’ R package was employed to generate forest plots for the univariate Cox regression analysis.

### Diagnostic value of ZNF692 in pan cancer

RNAseq data from the STAR process of 33 tumor projects obtained from TCGA were downloaded and collated. The data was extracted in TPM format and subjected to log2 transformation (value+1) for processing [19]. ROC analysis of the data was conducted using the pROC package, and the resulting outcomes were visually represented using ggplot2.

### The correlation between the expression of ZNF692 and staging in pan cancer

The GEPIA 2 website (http://gepia2.cancer-pku.cn/#index) was utilized to investigate the expression patterns of ZNF692 across various clinical stages of pan cancer [20].

### The correlation between the expression of ZNF692 and TMB/MSI in pan cancer

RNAseq data (level3) and corresponding clinical information for 33 tumors were obtained from the TCGA database. Additionally, TMB and MSI information were collected from the references [21, 22].

### Genomic alterations of ZNF692 in pan cancer

We investigated the genetic modifications of ZNF692 across multiple cancer types utilizing the cBioPortal platform (http://www.cbioportal.org/) [19].

### Correlation between ZNF692 expression and immune and immune checkpoints in pan cancer

The correlation between ZNF692 expression and cancer-associated fibroblast (CAF) infiltration was investigated in a pan-cancer context using the “immune” module of TIMER2, accessible at http://timer.cistrome.org/ [23].

RNAseq data (level3) and corresponding clinical information for 33 tumors were obtained from the TCGA database. The expression levels of eight immune checkpoint genes (SIGLEC15, IDO1, CD274, HAVCR2, PDCD1, CTLA4, LAG3, and PDCD1LG2) were examined [24].

### ZNF692-associated pathways in pan cancer

The proteins that interact with ZNF692 were examined through utilization of the STRING website (version 11.5, https://string-db.org). The top 100 genes exhibiting comparable expression patterns to ZNF692 across various cancer types were subjected to analysis using GEPIA2. Subsequently, an enrichment analysis of the aforementioned genes was conducted via the DAVID website (https://david.ncifcrf.gov/) [25, 26].

### Correlation analysis between the expression of ZNF692 and drug sensitivity

The RNAactDrug database (http://bio-bigdata.hrbmu.edu.cn/RNAactDrug/index.jsp) was employed to conduct an analysis on the association between ZNF692 expression and drug sensitivity across various types of cancer [11].

### Expression analysis of ZNF692 by single cell sequencing

CancerSEA (http://biocc.hrbmu.edu.cn/CancerSEA/home.jsp) was employed to investigate the association between ZNF692 expression and diverse functional states of tumors [27].

### Construction of ZNF692 mediated ceRNA network

In order to investigate the potential regulatory network of ZNF692 in HCC, we employed miRTarBase (http://mirtarbase.cuhk.edu.cn/) and TarBase V.8 (https://carolina.imis.athena-innovation.gr/diana_tools/web/index.php?r=tarbasev8%2Findex) databases to predict the miRNAs that interact with ZNF692 [11]. Subsequently, utilizing the miRNAs identified from the screening process, we utilized StarBase (http://starbase.sysu.edu.cn/) and LncBase Predicted v.2 (https://carolina.imis.athena-innovation.gr/diana_tools/web/index.php?r=lncbasev2/index-predicted) databases to predict the lncRNAs that bind to these miRNAs.

MiRNAseq data were obtained from level 3 BCGSC miRNA profiling, while RNAseq data in level 3 HTSeq-FPKM format were acquired from the TCGA HCC project [28, 29]. Statistical analysis and visualization were performed using R (version 3.6.3) and ggplot2 (version 3.3.3), respectively [29].

We collected miRNAseq data from level 3 BCGSC miRNA profiling and RNAseq data in level 3 HTSeq-FPKM format from the TCGA HCC project [25, 30]. Statistical analysis and visualization were conducted using R (version 3.6.3), along with the survminer package (version 0.4.9) and the survival package (version 3.2-10) [14]. We collected prognostic data from the references [31].

### QRT-PCR

The human normal liver epithelial cell line HepaRG, as well as the HCC cell lines HepG2, 97L, 97H, and HCCC-9810, were cultivated in our laboratory specifically for this study. HepaRG cells were cultured in 1640 medium (C11875500BT, Gibco, USA) supplemented with 10% fetal bovine serum (FBS). HepG2 were cultured with MEM (cat: M1003, Kinlogix, Canada) plus 10% FBS. 97L and 97H were cultured in DMEM high glucose (Gibco, C11965500BT) plus 10% (FBS). HCCC-9810 were cultured in RPMI 1640 (cat: M1002, Kinlogix, Canada) plus 10% FBS. We incubated the above cells at 5% CO_2_ and 37°C at rest. To identify ZNF692 levels in the HepG2, 97L, 97H, and HCCC-9810, qRT-PCR was performed according to the reference [32]. The primer sequences employed in this study were provided below: GAPDH-F: 5’-GAGTCAACGGATTTGGTCGT-3’, GAPDH-R: 5’-GACAAGCTTCCCGTTCTCAG-3’; ZNF692-F: 5’-TCTTTCCGCACTAGCAGCAA-3’, ZNF692-R: 5’-GGACCACTGGGTGACTCTTG-3’.

### Statistical analysis

Statistical analysis was conducted using R version 4.0.3, while a significance level of p < 0.05 was employed to determine statistically significant differences [17].

## Supplementary Material

Supplementary Table 1
