# Supplementary material for: Comprehensive analysis of ZNF692 as a potential biomarker associated with immune infiltration in a pan cancer analysis and validation in hepatocellular carcinoma
Source: Aging (Albany NY). 2023 Nov 17;15(22):13041–58. doi: 10.18632/aging.205218 (PMC10713400; doi:10.18632/aging.205218)
Supplement: Supplementary Table 1 [file aging-15-205218-s001.pdf]

SUPPLEMENTARY TABLE

Supplementary Table 1. Drug sensitivity analysis of ZNF692.

Compound	Source	Spearman.stat	Spearman.fdr	P-value
chlorobisoximatobismuth(iii)-dihydrochloride	CellMiner	0.653	0.000	1.54E-08
chlorotris(chinoline-8-olato)titanium(iv)-trihydrochloride	CellMiner	0.653	0.000	1.58E-08
tetrakis(8-chinolineolato)titanium(iv)-tetrahydrochloride	CellMiner	0.606	0.001	2.90E-07
benzoxazole, 2-[(1-methyl-4-nitro-1h-imidazol-5-yl)thio]-	CellMiner	0.605	0.001	3.12E-07
antineoplastic-637578	CellMiner	0.603	0.003	3.36796E-07
1-naphthalenecarboxamide, n,n'-1,8-(octanediyl)bis-	CellMiner	-0.497	0.002	<0.05
N-cyclohexyl-4-((1-phenyl-1H-pyrazolo[3,4-d]pyrimidin-4...	CellMiner	-0.508	0.003	3.39E-05
4-piperidinone, 3,5-dimethyl-1-nitroso-2,6-diphenyl-	CellMiner	-0.525	0.001	1.68E-05
(E)-5-chloro-3-((6-(4-chlorophenyl)-2-cyclopropylimidaz...	CellMiner	-0.544	0.001	6.97E-06
1,3-diphenyl-4-(3-phenyl-4,5-dihydro-1H-pyrazol-5-yl)-1...	CellMiner	-0.575	0.001	1.54E-06
